# Rapidly blinding posterior tubercular uveitis

**DOI:** 10.1186/1869-5760-4-13

**Published:** 2014-06-09

**Authors:** Manisha Agarwal, Vivek Jha, Jyotirmay Biswas

**Affiliations:** 1 Vitreoretina Services, Dr. Shroff’s Charity Eye Hospital, 5027-Kedar Nath Road, Daryaganj, New Delhi 110002, India; 2 Internal Medicine Services, Dr. Shroff’s Charity Eye Hospital, 5027-Kedar Nath Road, Daryaganj, New Delhi 110002, India; 3 Sankara Nethralaya, 18, College Road, Chennai, Tamil Nadu 600006, India

**Keywords:** Ocular tuberculosis, Blinding, Posterior

## Abstract

**Background:**

A 21-year-old female patient had chorioretinitis in the left eye which relapsed while being on anti-tubercular treatment and oral corticosteroids leading to blindness and the loss of the left eye.

**Findings:**

Mycobacterium tuberculosis causing chorioretinitis showed a poor response, and the lung lesions showed a good response to the same anti-tubercular treatment.

**Conclusions:**

Mycobacterium tubercle bacilli in the eye may show a poor response to the anti-tubercular drugs due to poor ocular penetration of the drugs secondary to early ocular hypoxia.

## Findings

### Introduction

Intraocular tuberculosis represents an extrapulmonary form of tuberculosis. Posterior uveitis is the most common manifestation of intraocular tuberculosis [1]. The inflammation predominantly involves the choroid in the form of choroidal tubercles, tuberculoma, sub-retinal abscess, chorioretinitis, and serpiginous-like choroiditis. We report a case of pulmonary and intraocular tuberculosis which showed a good response to the anti-tubercular drugs in the lungs but not in the eye leading to a blinding posterior tubercular uveitis.

## Case report

**Figure 1:**
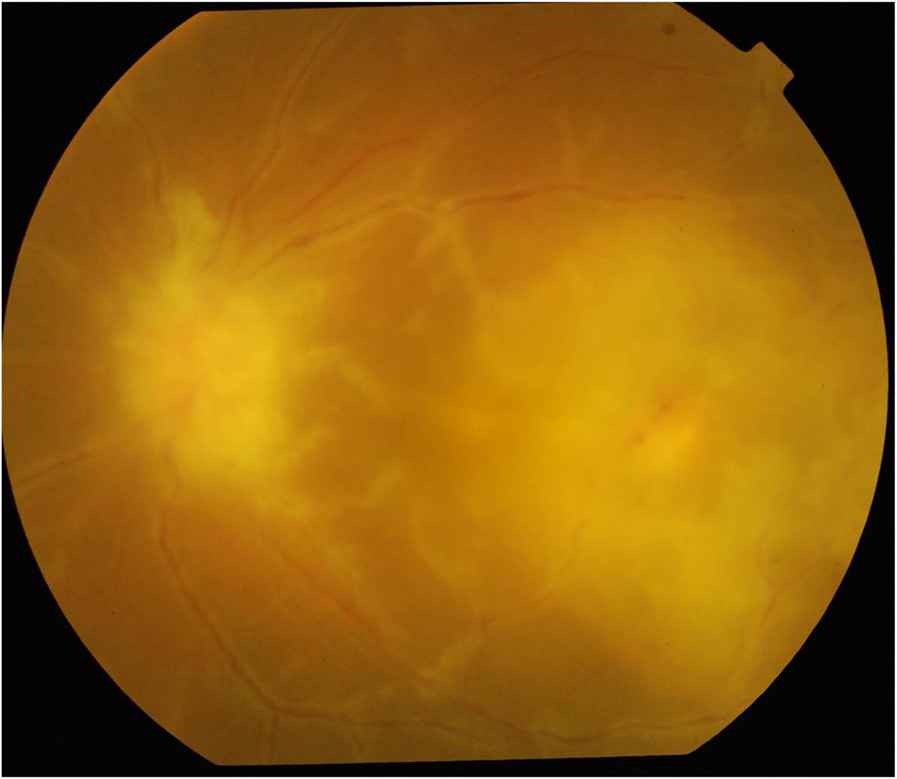
Granuloma on the ONH with perivascular sheathing and chorioretnitis patch with retinal hemorrhages in the macula.

A 21-year-old female presented with sudden painless diminution of vision in the left eye for 10 days. There was an associated history of low grade fever with evening rise and nonpurulent cough. There was a history of pulmonary tuberculosis in her brother. On examination, the best corrected visual acuity (BCVA) was 20/20, N6 in the right eye and counting finger close to face in the left eye. Slit lamp examination was unremarkable in both eyes. Fundus examination of the right eye was normal, and the left eye showed a granuloma on the optic nerve head (ONH) with perivascular sheathing and chorioretinitis patch with overlying retinal hemorrhages in the macula (Figure 1).

**Figure 2:**
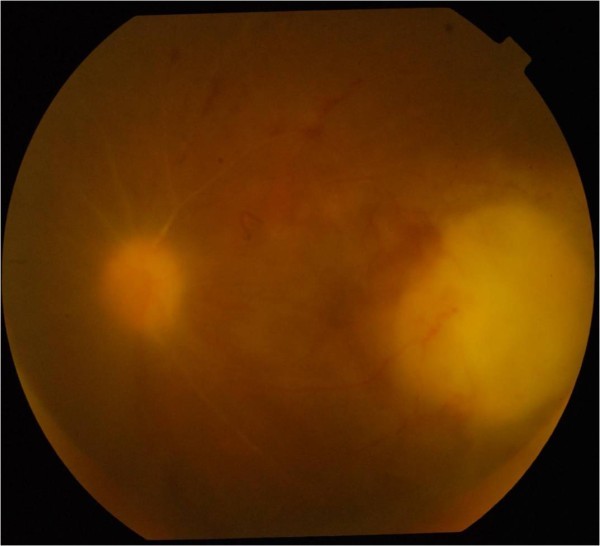
The chorioretinitis patch in the macula decreased with a decrease in the granuloma on the disc and perivasculitis.

**Figure 3:**
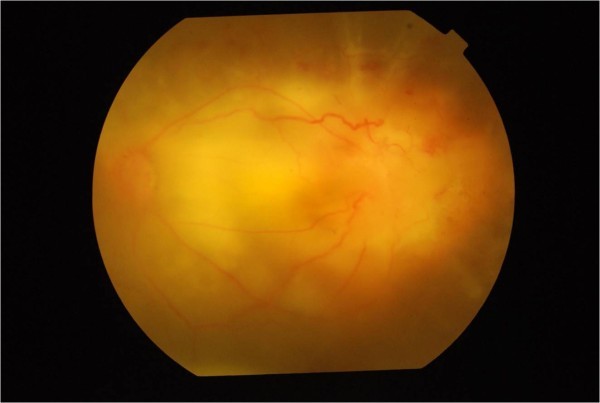
Appearance of new patches of chorioretinitis in the macula.

Blood investigation showed hemoglobin 7.6 gm%, raised erythrocyte sedimentation rate (ESR) 65 mm in the first hour, and Mantoux test -26 × 26 mm. Sputum test was negative for acid fast bacilli. Chest X-ray was normal and a high-resolution computed tomography (HRCT)-thorax scan showed enlarged mediastinal lymph nodes with necrosis in the center and patches of infiltration in the left apical region and right lower lobe. She was evaluated by the pulmonologist and started on four-drug regimen of anti-tubercular therapy (ATT) comprising of rifampicin 600 mg/day, ethambutol, pyrazinamide and isoniazid, and systemic corticosteroids 1 mg/kg body weight. Topical prednisolone 1% eye drops and atropine 1% were also started. There was evidence of mild vitritis after 1 week on treatment.After 1 month follow-up, the BCVA of the left eye was counting fingers at 2 meters and fundus examination showed a decrease in the size of the chorioretinitis patch in the macula with a decrease in the paerivasculitis and the granuloma on the ONH (Figure 2).Two weeks later, she presented with severe pain in the left eye. BCVA in the left eye was counting fingers at 2 meters and fundus examination of the left eye showed appearance of new patches of chorioretinitis in the macula between the ONH and previous patch of chorioretinitis (Figure 3). Systemic evaluation and repeat HRCT-thorax showed resolution of the chest infiltrates. The systemic corticosteroids were increased to 1.5 mg/kg/body weight and oral ofloxacin 400 mg twice daily was added to the ongoing four-drug ATT.

**Figure 4:**
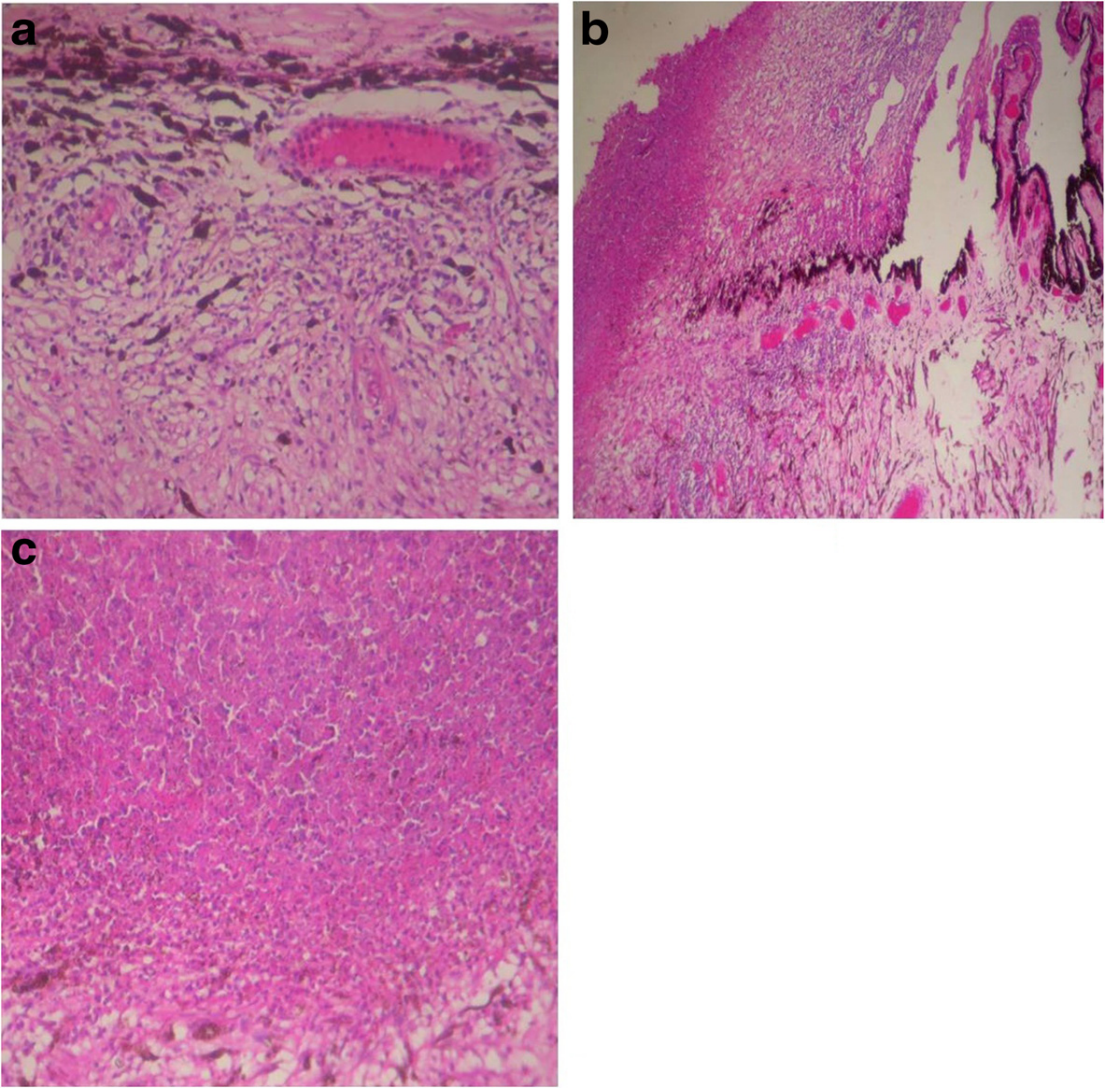
Microscopic examination of the sub-retinal areas (a,b,c).

**Figure 5:**
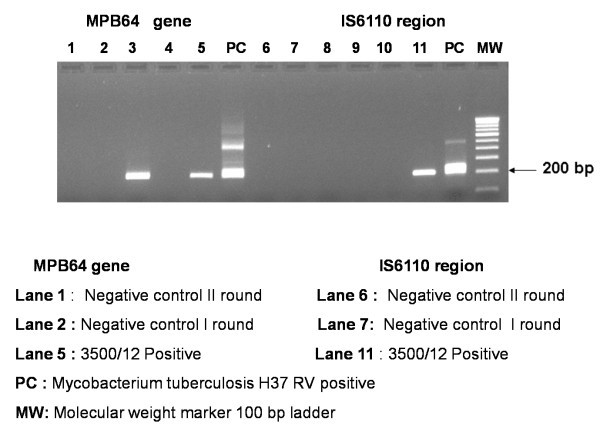
Positive polymerase chain reaction for mycobacterium tuberculosis.

Follow-up after 2 weeks showed a decrease of vision in the left eye to perception of light with extensive neovascularization of the iris (NVI), posterior synechiae formation, and early lenticular changes. The intraocular pressure (IOP) by Goldmann applanation tonometer was 28 mm of Hg. Ultrasound B-scan showed exudative retinal detachment with evidence of choroidal thickening. She was treated with off-label use of intravitreal injections of bevacizumab (Avastin, Genentech, South San Francisco, CA, USA). The left eye subsequently became a painful blind eye which was digitally soft with a decreased axial length on ultrasound B-scan.Enucleation with ball implant was done for the left eye after an informed consent. Gross histopathology of the left eyeball showed total retinal detachment with choroidal thickening. Microscopic examination of the sub-retinal areas showed extensive caseation necrosis with intraretinal inflammation and dense collections of lymphocytes and plasma cells, choroidal inflammation with lymphocytic cells, chronic inflammation of the ciliary body, and inflammation of the scleral fibers (Figure 4a,b,c).On microbiological examination, acid fast bacilli (AFB) stain was negative for mycobacterium tubercle bacilli. Grams’ stain was negative for fungal filaments. Polymerase chain reaction (PCR) for mycobacterium tuberculosis was positive with both the primers IS6110 and MPb 64 (Figure 5).

She was subsequently dispensed with a prosthetic shell for the left socket.

## Discussion

Tuberculosis is a clinical disease caused by infection with *Mycobacterium tuberculosis* and is characterized pathologically by the formation of granulomas [2]. It is commonly described as a systemic disease of ‘protean manifestations’ that mainly involves the lungs. Extrapulmonary involvement, including lesions of the gastrointestinal tract, genito-urinary tract, cardiovascular system, skin, central nervous system, and eyes, may occur either in association with clinically apparent pulmonary tuberculosis or in isolation, with no clinical or laboratory evidence of pulmonary infection. In India, where pulmonary tuberculosis is endemic, the incidence of ocular involvement is variable [3].

In our patient, there was initially a response to the oral corticosteroids and four-drug ATT; however, this was 2 weeks later followed by a deterioration in the left eye and improvement in the lung lesions while being on treatment. We got different response in the lungs and in the eye even though it is presumed that the strain of mycobacterium tubercle bacilli causing infection in the lungs would also be the causative bacilli in the left eye. This excludes the fact that our case was of a multidrug-resistant tuberculosis as then neither lungs or the eye would have responded to the treatment. There was a resistance to start second line anti-tubercular drugs seeing to the resolution of the chest lesions; however, oral ofloxacin was added which failed to provide any additional benefit. We were unable to see the antibiotic sensitivity of the mycobacterium tubercle bacilli as the microbiology testing showed no growth though the polymerase chain reaction confirmed the same.

This case is reported to highlight the fact that it is not essential that pulmonary and extrapulmonary manifestation especially ocular involvement of tuberculosis may show the same response to the ATT. This maybe secondary to the fact that ocular hypoxia sets in very fast in eyes with inflammation of the choroid secondary to mycobacterium tuberculosis [4-6]. This causes decreased choroidal circulation, and this may reduce the penetration of the drugs of ATT into the eye. Early neovascular complications are seen in these eyes secondary to ocular hypoxia as in our case where we treated our patient with multiple intravitreal injections of bevacizumab (Avastin,Genentech) which is a nonselective vascular endothelial growth factor inhibitor providing relief from pain till the eye was enucleated. We need to be very careful and follow a case of intraocular tuberculosis at frequent intervals as there maybe a very fast progression of ocular tuberculosis leading to a complete loss of the eye.

## Abbreviations

AFB: acid fast bacilli; ATT: anti-tubercular treatment; BCVA: best corrected visual acuity; ESR: erythrocyte sedimentation rate; HRCT: high-resolution computed tomography; IOP: intraocular pressure; NVI: neovascularization of iris; ONH: optic nerve head; PCR: polymerase chain reaction.

## Competing interests

The authors declare that they have no competing interests.

## Authors’ contributions

MA provided ophthalmic care to the patient and drafted the manuscript. VJ managed the patient systemically and monitored the anti-tubercular treatment. JB did the histopathological examinations. All the authors read and approved the final manuscript.

## Authors’ information

Dr. Manisha Agarwal, M.S. (Ophthalmology), is the head of Vitreoretina Services, Dr.Shroff’s Charity Eye Hospital, New Delhi, India. Dr. Vivek Jha, M.D. (Medicine), is a physician and pulmonologist at Dr. Shroff’s Charity Eye Hospital, New Delhi, India. Dr. Jyotirmay Biswas, M.S. (Ophthalmology) is the head of Uvea and Ocular Pathology, Sankara Nethralaya, Chennai, India.
